# Retraction Note: Analysis of the factors influencing moderate to poor performance status in patients with cancer after chemotherapy: a cross-sectional study comparing three models

**DOI:** 10.1038/s41598-026-55887-x

**Published:** 2026-06-03

**Authors:** Ke Xi, Lin Jingping, Liu Yaqing, Yu Xinyuan, Lin Hui, Yang Mei, Chen Qingyue, Liu Dun

**Affiliations:** 1 Nursing Department, Clinical Oncology School of Fujian Medical University, Fujian Cancer Hospital, Fuzhou, 350014 Fujian China; 2 Department of Critical Care Medicine, Clinical Oncology School of Fujian Medical University, Fujian Cancer Hospital, Fuzhou, 350014 Fujian China; 3 The School of Nursing, Fujian Medical University, Fuzhou, 350122 Fujian Province China; 4 Department of Abdominal Oncology, Clinical Oncology School of Fujian Medical University, Fujian Cancer Hospital, Fuzhou, 350014 Fujian China; 5 Nursing School, Fujian Medical University, No. 1, Xuefu North Road, Shangjie Town, Minhou County, Fuzhou City, 350014 Fujian Province China

Retraction of: *Scientific Reports* 10.1038/s41598-024-53481-7, published online 09 February 2024

The Editors have retracted this Article.

After publication of the Article, concerns were raised about an extremely high AUC of the prediction model as well as discrepancies between the methods and results. Upon request, the Authors could not provide a satisfactory response to these concerns. In addition, an investigation by the Editors found inconsistencies between the raw data provided by the Authors and the results presented in the Article. The Editors therefore no longer have confidence in the reliability of the content of this Article.

All Authors disagree with this retraction.

